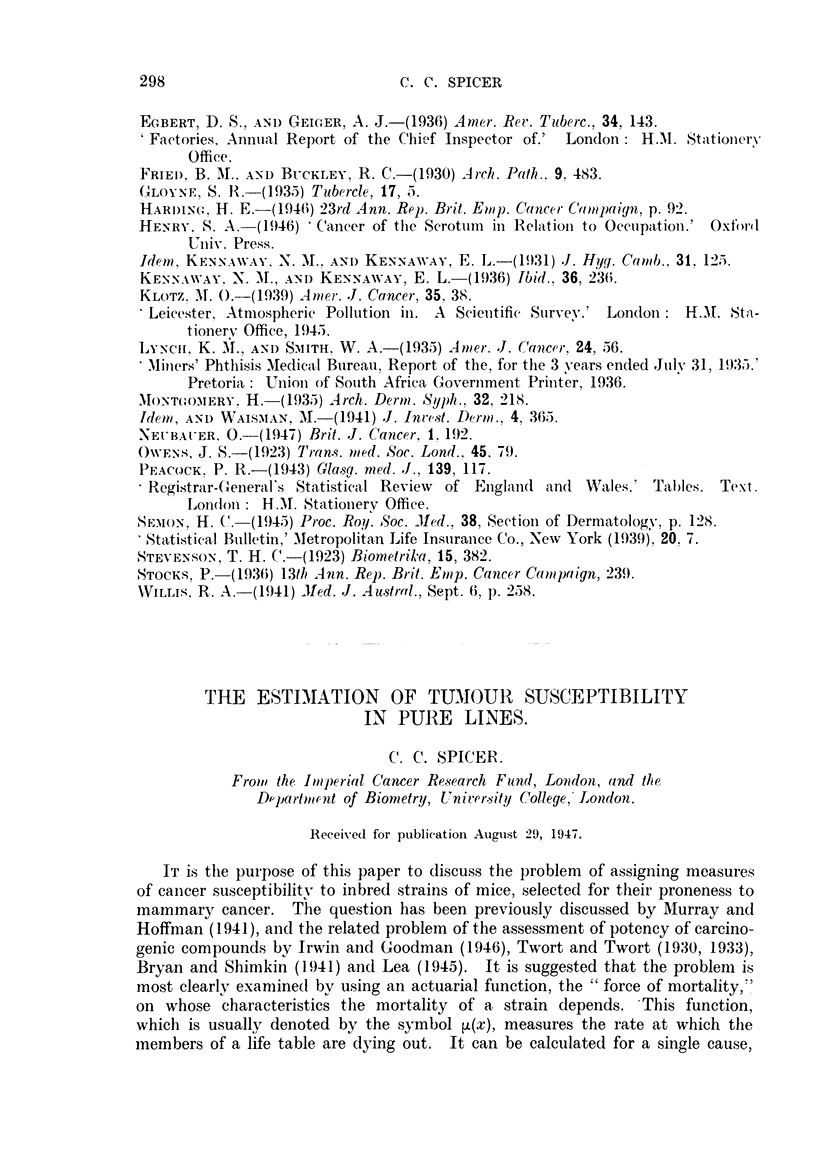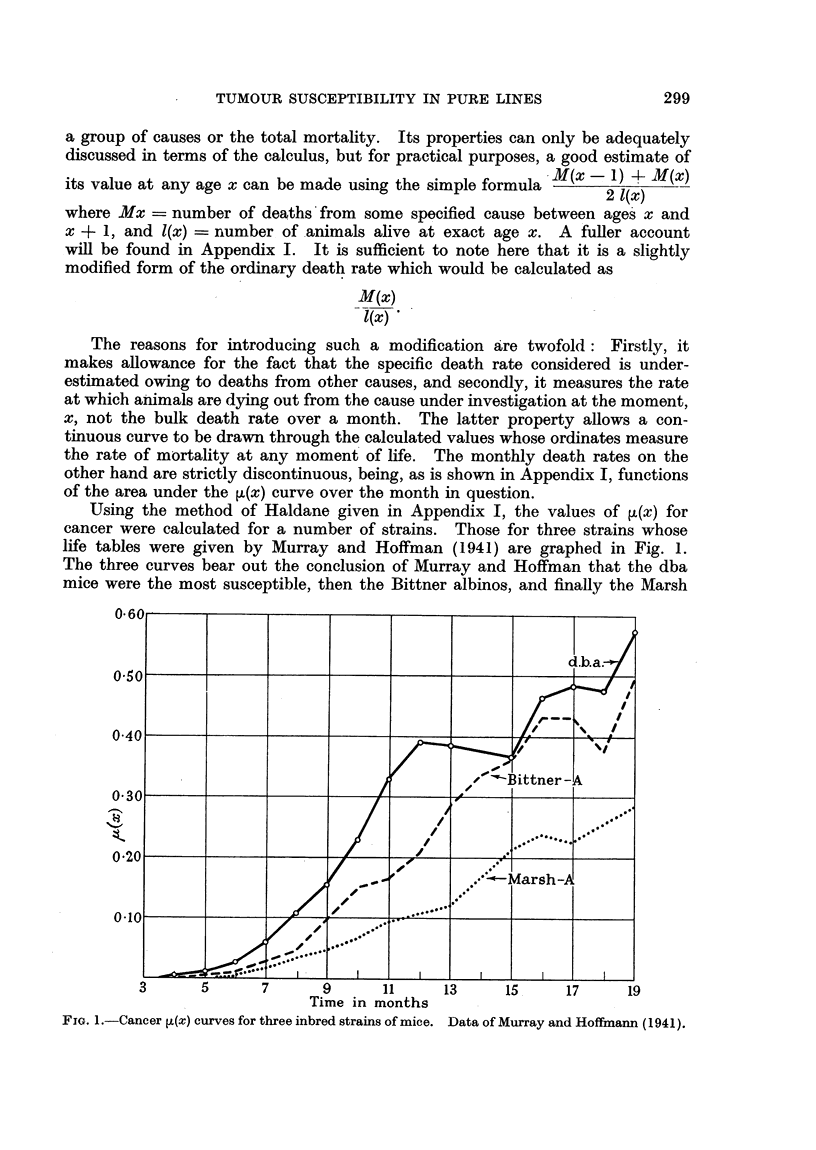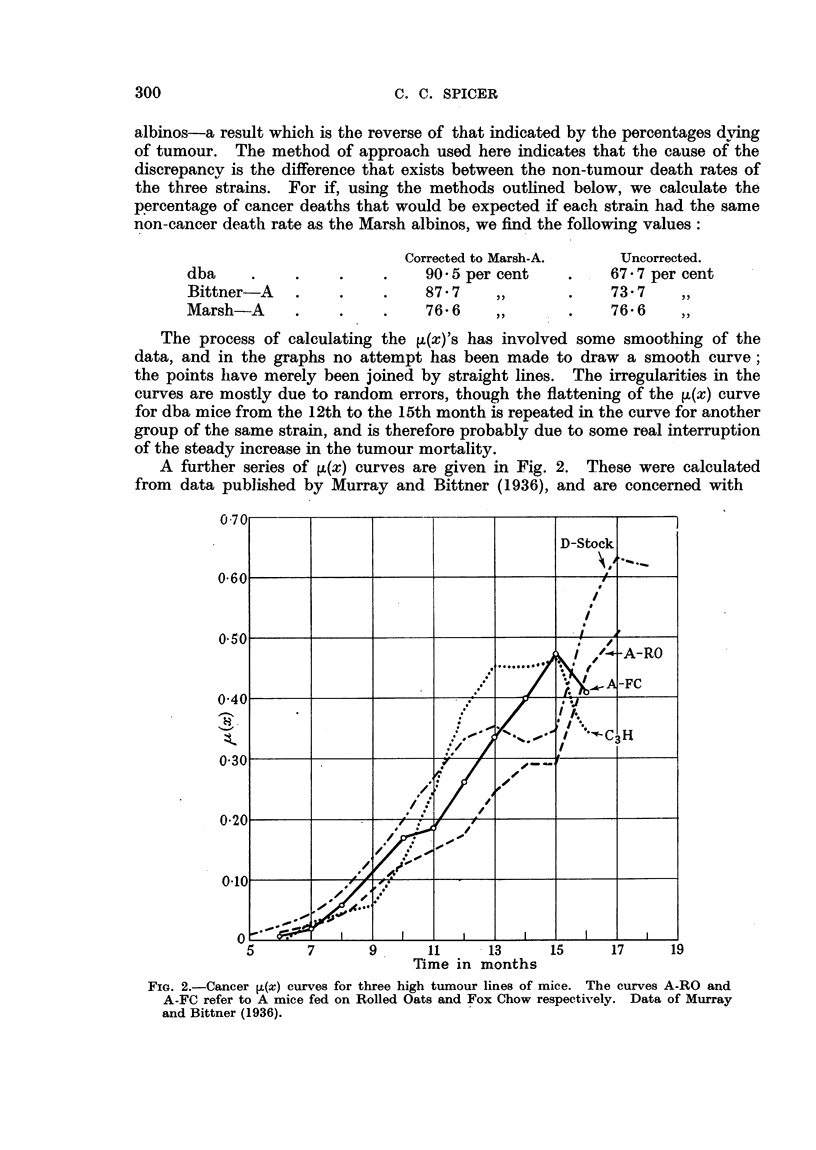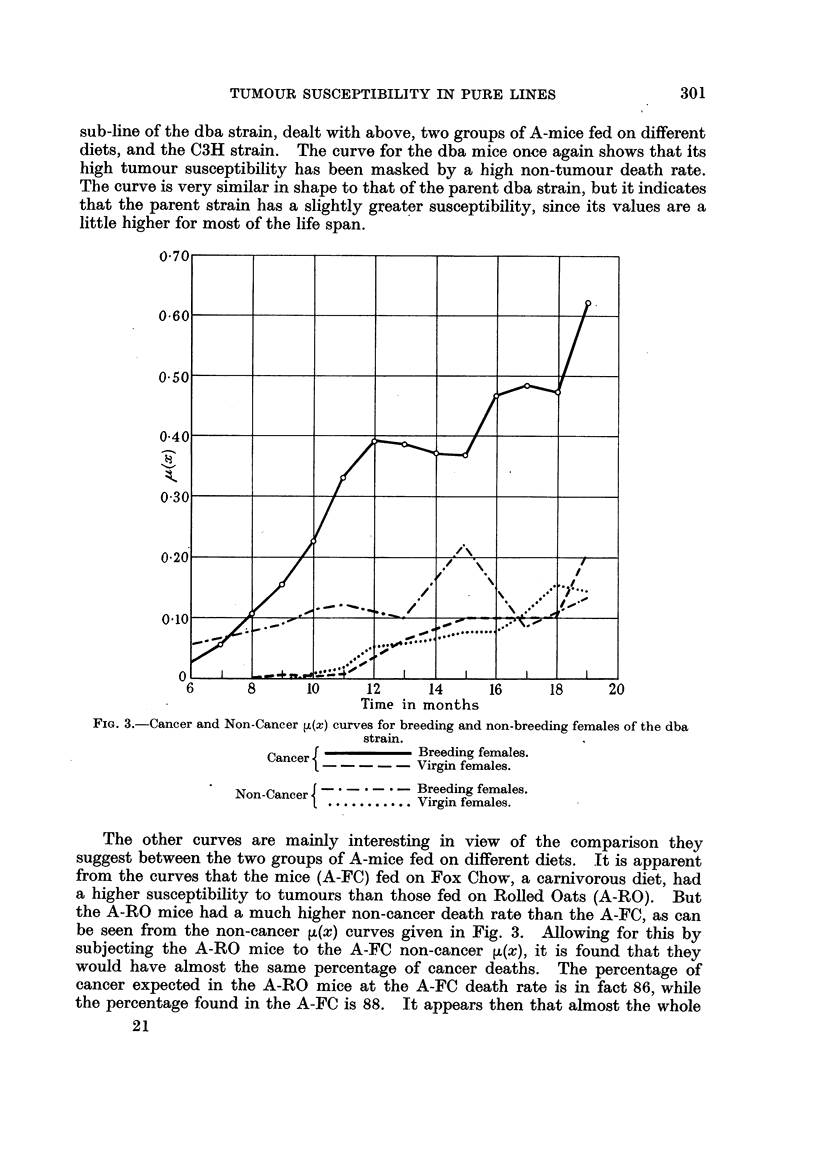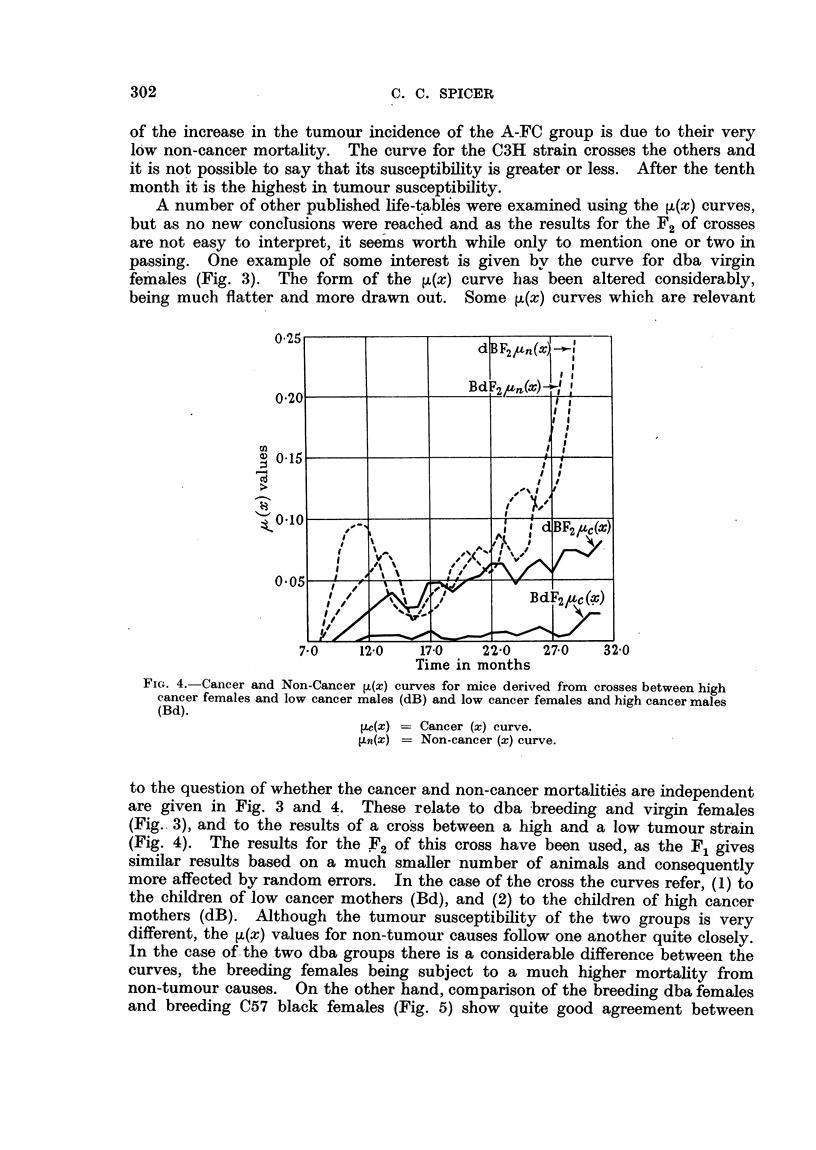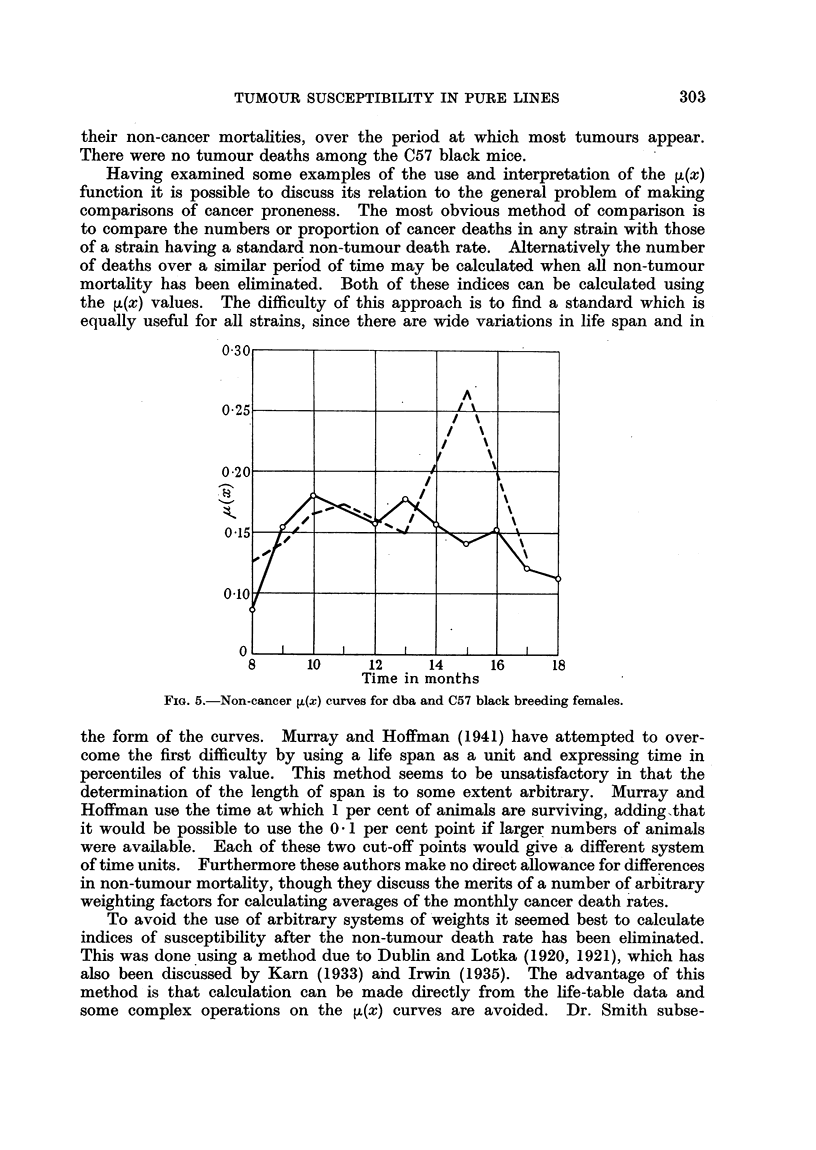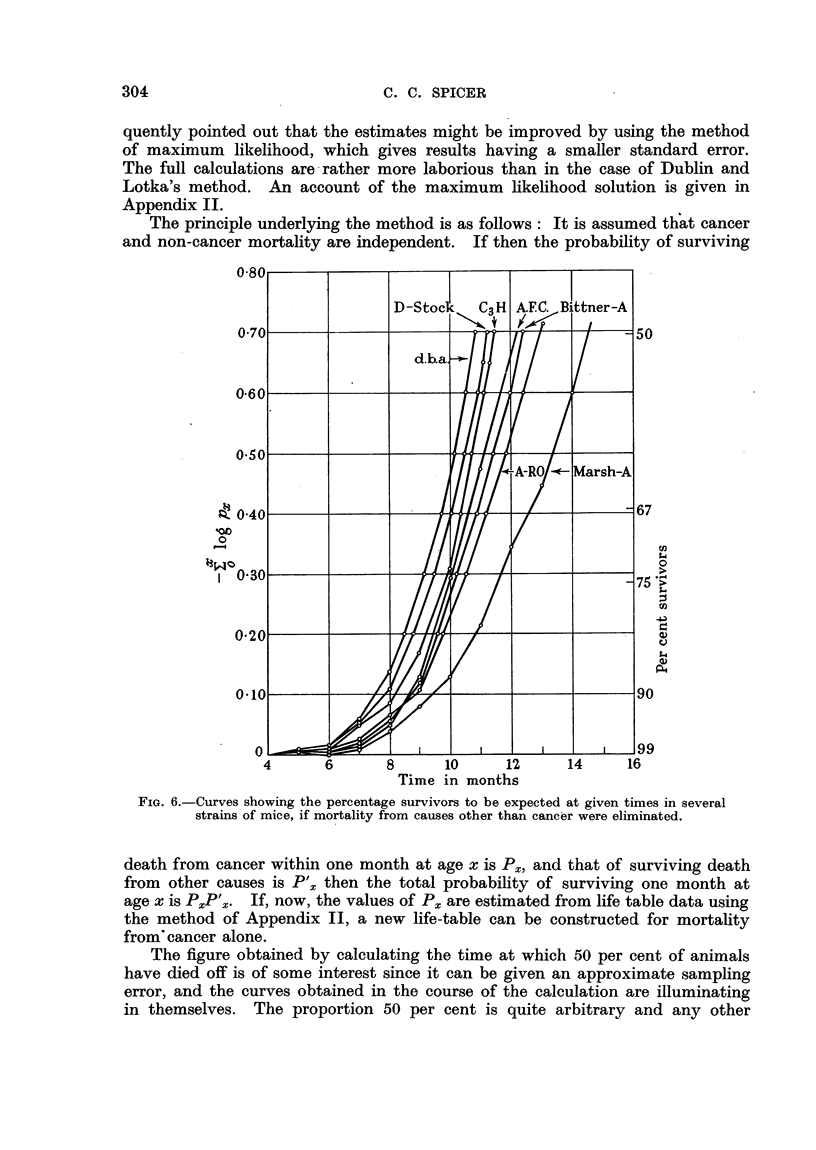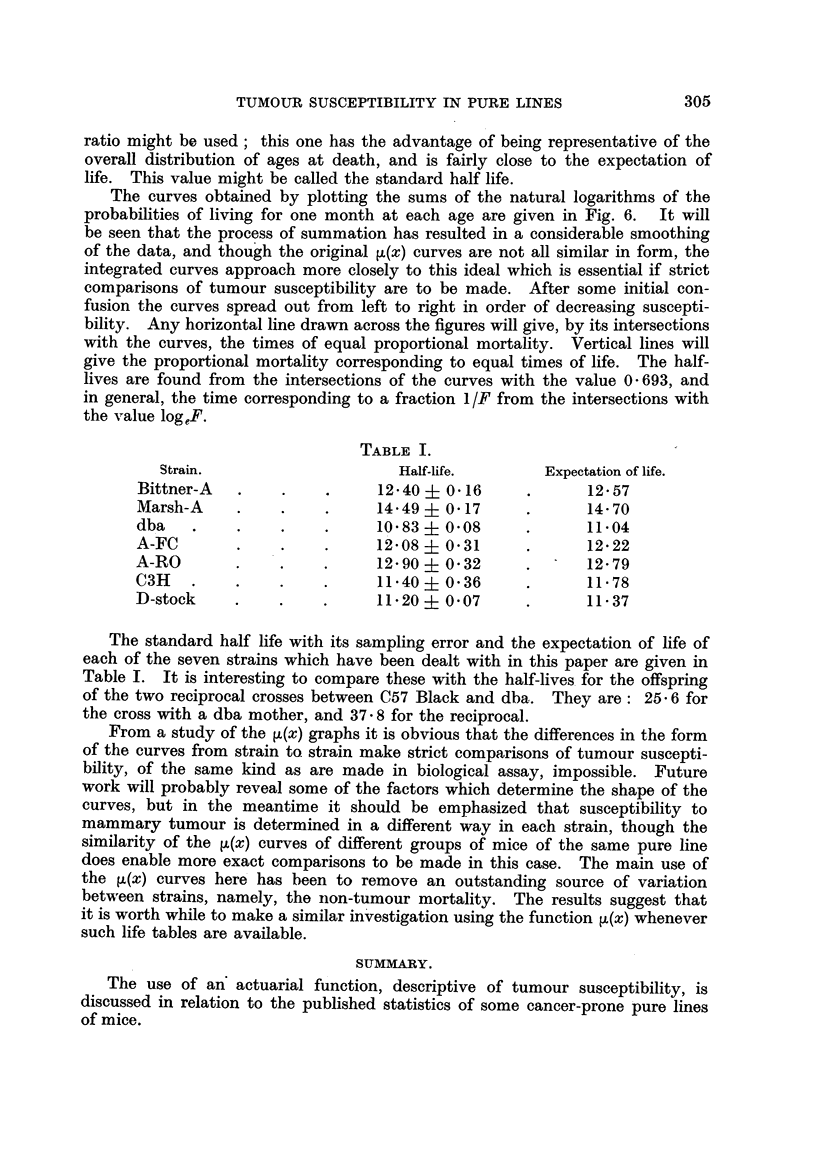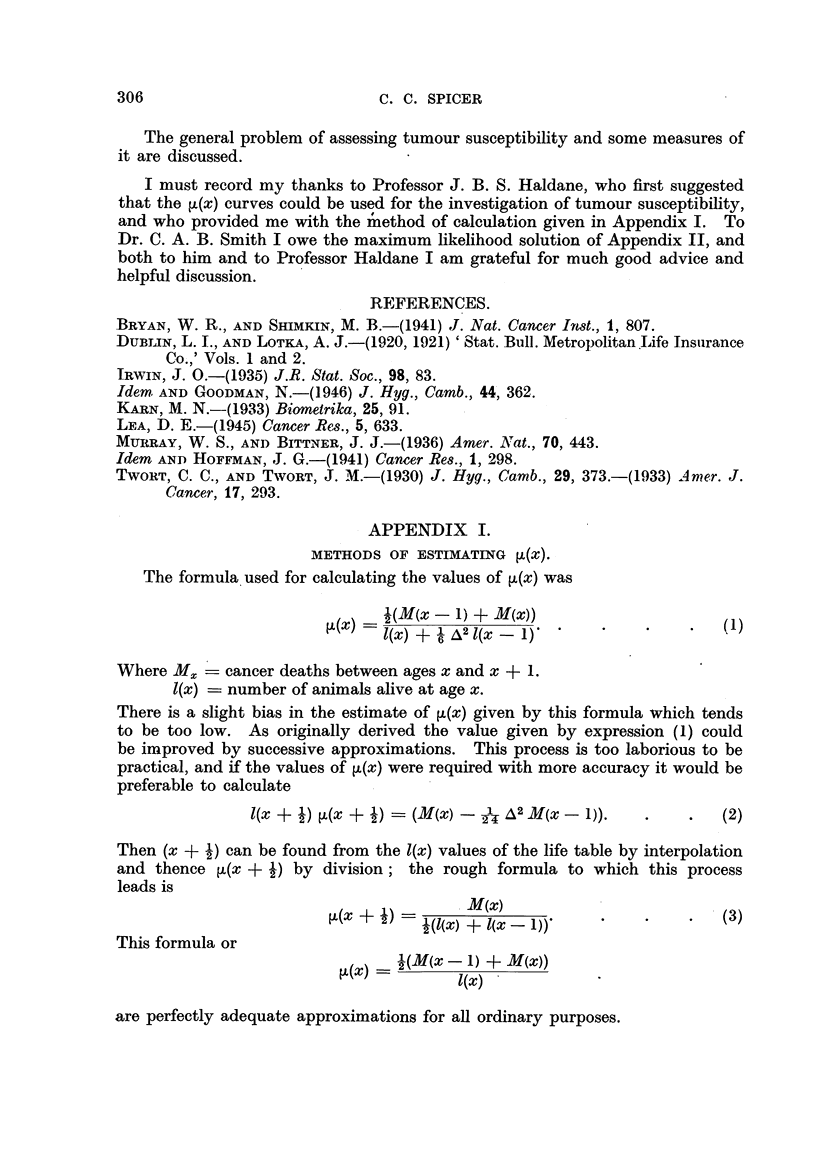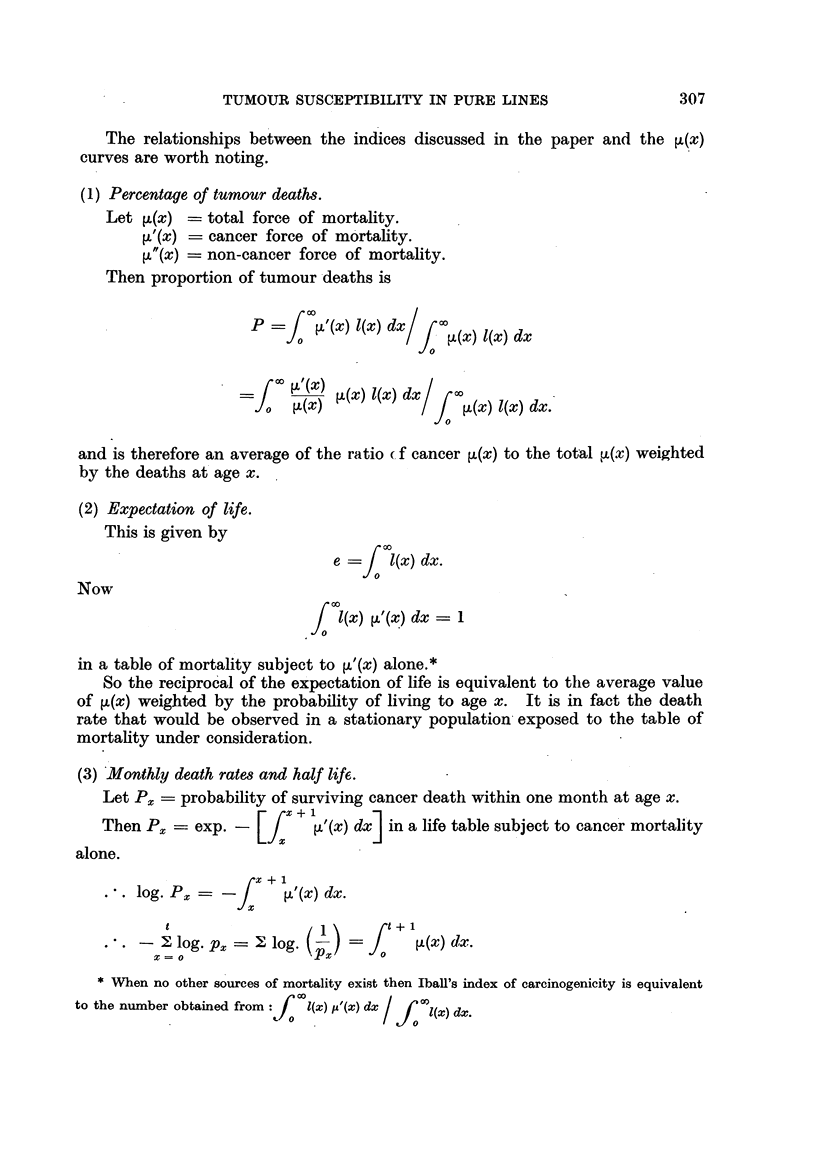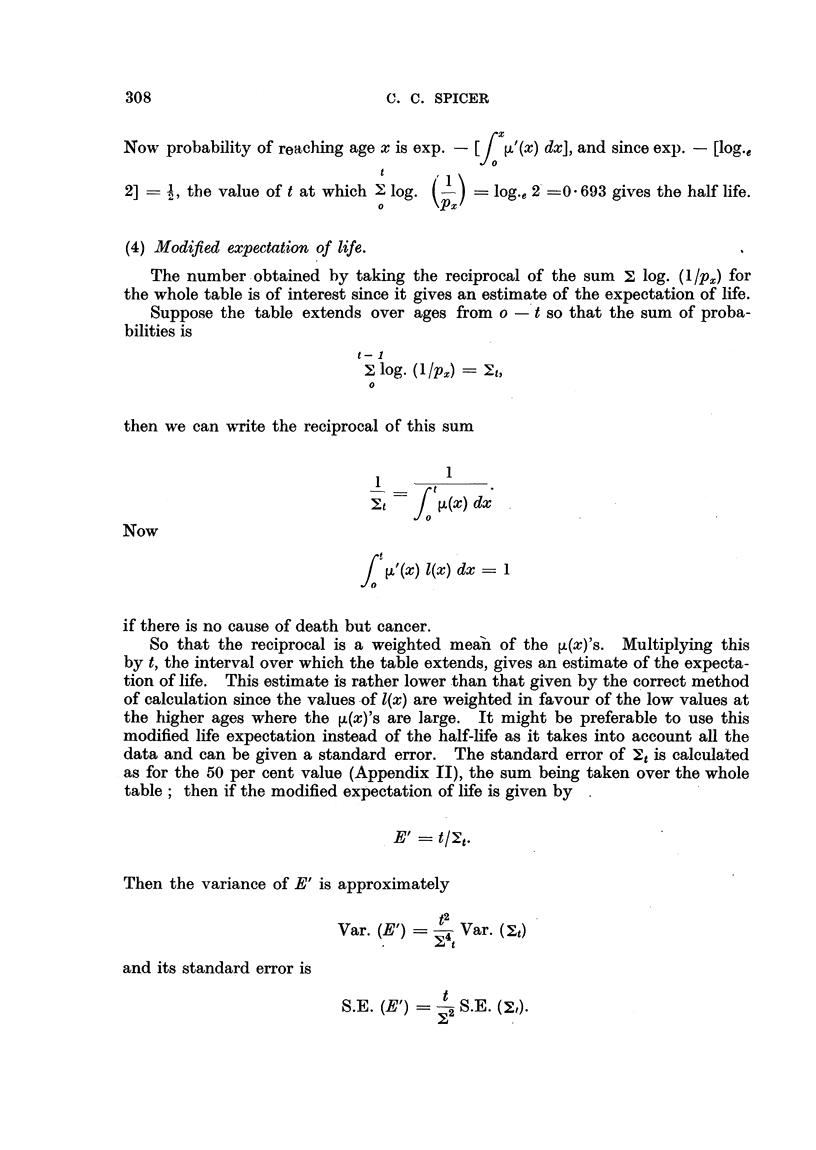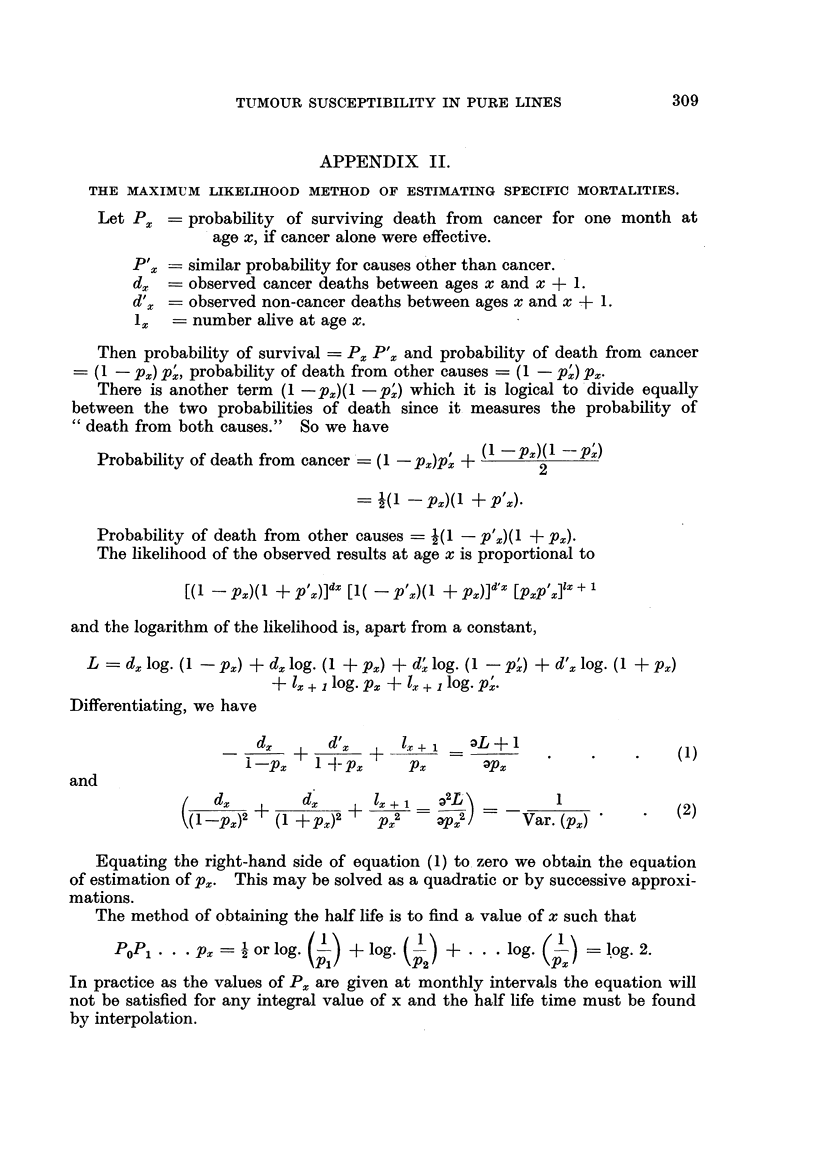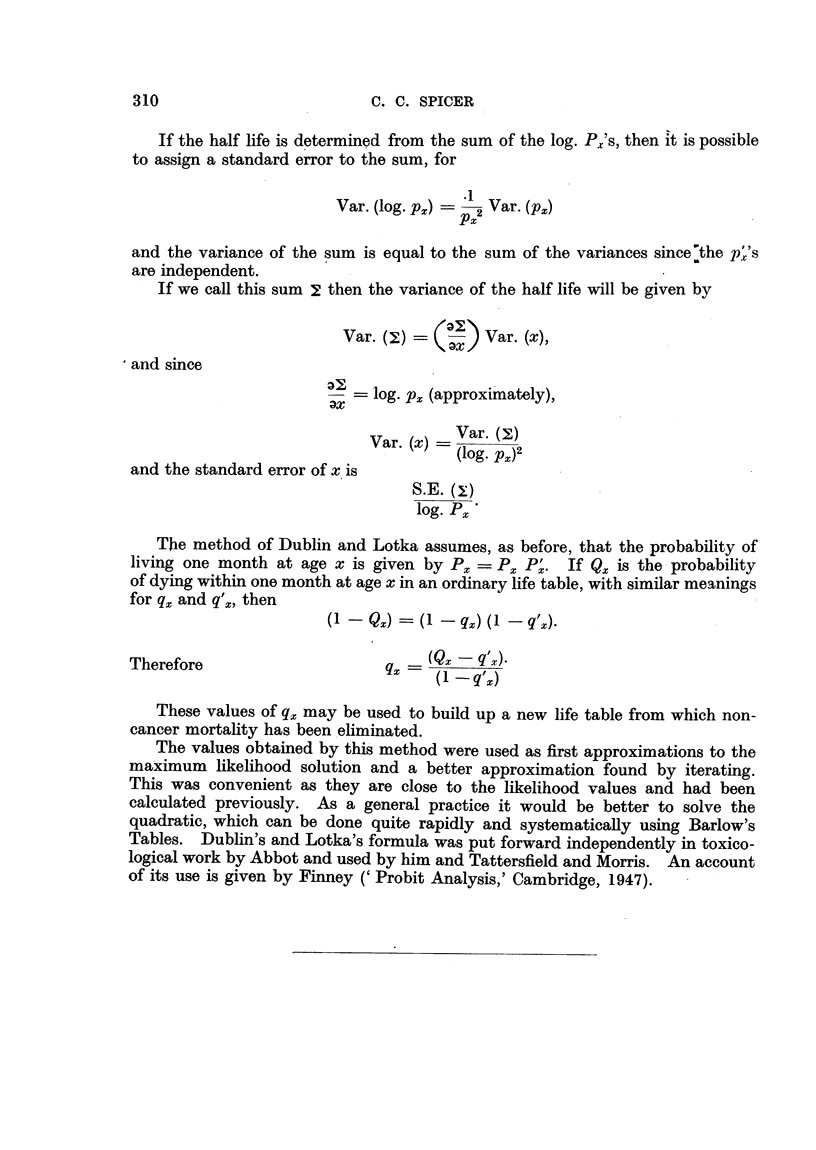# The Estimation of Tumour Susceptibility in Pure Lines

**DOI:** 10.1038/bjc.1947.25

**Published:** 1947-09

**Authors:** C. C. Spicer


### THE ESTIMATION OF TUMOUR SUSCEPTIBILITY IN PURE LINES.

C. C. SPICER.

*From the Imperial Cancer Research Fund, London, and the Department of Biometry, University College, London.*

Received for publication August 29, 1947.

IT is the purpose of this paper to discuss the problem of assigning measures of cancer susceptibility to inbred strains of mice, selected for their proneness to mammary cancer. The question has been previously discussed by Murray and Hoffman (1941), and the related problem of the assessment of potency of carcinogenic compounds by Irwin and Goodman (1946), Twort and Twort (1930, 1933), Bryan and Shimkin (1941) and Lea (1945). It is suggested that the problem is most clearly examined by using an actuarial function, the "force of mortality," on whose characteristics the mortality of a strain depends. This function, which is usually denoted by the symbol $\mu(x)$, measures the rate at which the members of a life table are dying out. It can be calculated for a single cause, a group of causes or the total mortality. Its properties can only be adequately discussed in terms of the calculus, but for practical purposes, a good estimate of its value at any age $x$ can be made using the simple formula $\frac{M(x-1)+M(x)}{2\,l(x)}$ where $Mx$ = number of deaths from some specified cause between ages $x$ and $x+1$, and $l(x)$ = number of animals alive at exact age $x$. A fuller account will be found in Appendix I. It is sufficient to note here that it is a slightly modified form of the ordinary death rate which would be calculated as

$$\frac{M(x)}{l(x)}.$$

The reasons for introducing such a modification are twofold: Firstly, it makes allowance for the fact that the specific death rate considered is underestimated owing to deaths from other causes, and secondly, it measures the rate at which animals are dying out from the cause under investigation at the moment, $x$, not the bulk death rate over a month. The latter property allows a continuous curve to be drawn through the calculated values whose ordinates measure the rate of mortality at any moment of life. The monthly death rates on the other hand are strictly discontinuous, being, as is shown in Appendix I, functions of the area under the $\mu(x)$ curve over the month in question.

Using the method of Haldane given in Appendix I, the values of $\mu(x)$ for cancer were calculated for a number of strains. Those for three strains whose life tables were given by Murray and Hoffman (1941) are graphed in Fig. 1. The three curves bear out the conclusion of Murray and Hoffman that the dba mice were the most susceptible, then the Bittner albinos, and finally the Marsh

FIG. 1.—Cancer $\mu(x)$ curves for three inbred strains of mice. Data of Murray and Hoffmann (1941).

albinos—a result which is the reverse of that indicated by the percentages dying of tumour. The method of approach used here indicates that the cause of the discrepancy is the difference that exists between the non-tumour death rates of the three strains. For if, using the methods outlined below, we calculate the percentage of cancer deaths that would be expected if each strain had the same non-cancer death rate as the Marsh albinos, we find the following values :

| | Corrected to Marsh-A. | Uncorrected. |
|---|---|---|
| dba | 90·5 per cent | 67·7 per cent |
| Bittner—A | 87·7 ,, | 73·7 ,, |
| Marsh—A | 76·6 ,, | 76·6 ,, |

The process of calculating the $\mu(x)$'s has involved some smoothing of the data, and in the graphs no attempt has been made to draw a smooth curve ; the points have merely been joined by straight lines. The irregularities in the curves are mostly due to random errors, though the flattening of the $\mu(x)$ curve for dba mice from the 12th to the 15th month is repeated in the curve for another group of the same strain, and is therefore probably due to some real interruption of the steady increase in the tumour mortality.

A further series of $\mu(x)$ curves are given in Fig. 2. These were calculated from data published by Murray and Bittner (1936), and are concerned with

Fig. 2.—Cancer $\mu(x)$ curves for three high tumour lines of mice. The curves A-RO and A-FC refer to A mice fed on Rolled Oats and Fox Chow respectively. Data of Murray and Bittner (1936).

sub-line of the dba strain, dealt with above, two groups of A-mice fed on different diets, and the C3H strain. The curve for the dba mice once again shows that its high tumour susceptibility has been masked by a high non-tumour death rate. The curve is very similar in shape to that of the parent dba strain, but it indicates that the parent strain has a slightly greater susceptibility, since its values are a little higher for most of the life span.

FIG. 3.—Cancer and Non-Cancer $\mu(x)$ curves for breeding and non-breeding females of the dba strain.

Cancer { ———— Breeding females. / — — — — Virgin females.

Non-Cancer { — · — · — · — Breeding females. / . . . . . . . . . . . Virgin females.

The other curves are mainly interesting in view of the comparison they suggest between the two groups of A-mice fed on different diets. It is apparent from the curves that the mice (A-FC) fed on Fox Chow, a carnivorous diet, had a higher susceptibility to tumours than those fed on Rolled Oats (A-RO). But the A-RO mice had a much higher non-cancer death rate than the A-FC, as can be seen from the non-cancer $\mu(x)$ curves given in Fig. 3. Allowing for this by subjecting the A-RO mice to the A-FC non-cancer $\mu(x)$, it is found that they would have almost the same percentage of cancer deaths. The percentage of cancer expected in the A-RO mice at the A-FC death rate is in fact 86, while the percentage found in the A-FC is 88. It appears then that almost the whole of the increase in the tumour incidence of the A-FC group is due to their very low non-cancer mortality. The curve for the C3H strain crosses the others and it is not possible to say that its susceptibility is greater or less. After the tenth month it is the highest in tumour susceptibility.

A number of other published life-tables were examined using the $\mu(x)$ curves, but as no new conclusions were reached and as the results for the $F_2$ of crosses are not easy to interpret, it seems worth while only to mention one or two in passing. One example of some interest is given by the curve for dba virgin females (Fig. 3). The form of the $\mu(x)$ curve has been altered considerably, being much flatter and more drawn out. Some $\mu(x)$ curves which are relevant

FIG. 4.—Cancer and Non-Cancer $\mu(x)$ curves for mice derived from crosses between high cancer females and low cancer males (dB) and low cancer females and high cancer males (Bd).

$\mu_c(x)$ = Cancer ($x$) curve.

$\mu_n(x)$ = Non-cancer ($x$) curve.

to the question of whether the cancer and non-cancer mortalities are independent are given in Fig. 3 and 4. These relate to dba breeding and virgin females (Fig. 3), and to the results of a cross between a high and a low tumour strain (Fig. 4). The results for the $F_2$ of this cross have been used, as the $F_1$ gives similar results based on a much smaller number of animals and consequently more affected by random errors. In the case of the cross the curves refer, (1) to the children of low cancer mothers (Bd), and (2) to the children of high cancer mothers (dB). Although the tumour susceptibility of the two groups is very different, the $\mu(x)$ values for non-tumour causes follow one another quite closely. In the case of the two dba groups there is a considerable difference between the curves, the breeding females being subject to a much higher mortality from non-tumour causes. On the other hand, comparison of the breeding dba females and breeding C57 black females (Fig. 5) show quite good agreement between their non-cancer mortalities, over the period at which most tumours appear. There were no tumour deaths among the C57 black mice.

Having examined some examples of the use and interpretation of the $\mu(x)$ function it is possible to discuss its relation to the general problem of making comparisons of cancer proneness. The most obvious method of comparison is to compare the numbers or proportion of cancer deaths in any strain with those of a strain having a standard non-tumour death rate. Alternatively the number of deaths over a similar period of time may be calculated when all non-tumour mortality has been eliminated. Both of these indices can be calculated using the $\mu(x)$ values. The difficulty of this approach is to find a standard which is equally useful for all strains, since there are wide variations in life span and in

FIG. 5.—Non-cancer $\mu(x)$ curves for dba and C57 black breeding females.

the form of the curves. Murray and Hoffman (1941) have attempted to overcome the first difficulty by using a life span as a unit and expressing time in percentiles of this value. This method seems to be unsatisfactory in that the determination of the length of span is to some extent arbitrary. Murray and Hoffman use the time at which 1 per cent of animals are surviving, adding that it would be possible to use the 0·1 per cent point if larger numbers of animals were available. Each of these two cut-off points would give a different system of time units. Furthermore these authors make no direct allowance for differences in non-tumour mortality, though they discuss the merits of a number of arbitrary weighting factors for calculating averages of the monthly cancer death rates.

To avoid the use of arbitrary systems of weights it seemed best to calculate indices of susceptibility after the non-tumour death rate has been eliminated. This was done using a method due to Dublin and Lotka (1920, 1921), which has also been discussed by Karn (1933) and Irwin (1935). The advantage of this method is that calculation can be made directly from the life-table data and some complex operations on the $\mu(x)$ curves are avoided. Dr. Smith subsequently pointed out that the estimates might be improved by using the method of maximum likelihood, which gives results having a smaller standard error. The full calculations are rather more laborious than in the case of Dublin and Lotka's method. An account of the maximum likelihood solution is given in Appendix II.

The principle underlying the method is as follows : It is assumed that cancer and non-cancer mortality are independent. If then the probability of surviving

FIG. 6.—Curves showing the percentage survivors to be expected at given times in several strains of mice, if mortality from causes other than cancer were eliminated.

death from cancer within one month at age $x$ is $P_x$, and that of surviving death from other causes is $P'_x$ then the total probability of surviving one month at age $x$ is $P_xP'_x$. If, now, the values of $P_x$ are estimated from life table data using the method of Appendix II, a new life-table can be constructed for mortality from cancer alone.

The figure obtained by calculating the time at which 50 per cent of animals have died off is of some interest since it can be given an approximate sampling error, and the curves obtained in the course of the calculation are illuminating in themselves. The proportion 50 per cent is quite arbitrary and any other ratio might be used ; this one has the advantage of being representative of the overall distribution of ages at death, and is fairly close to the expectation of life. This value might be called the standard half life.

The curves obtained by plotting the sums of the natural logarithms of the probabilities of living for one month at each age are given in Fig. 6. It will be seen that the process of summation has resulted in a considerable smoothing of the data, and though the original $\mu(x)$ curves are not all similar in form, the integrated curves approach more closely to this ideal which is essential if strict comparisons of tumour susceptibility are to be made. After some initial confusion the curves spread out from left to right in order of decreasing susceptibility. Any horizontal line drawn across the figures will give, by its intersections with the curves, the times of equal proportional mortality. Vertical lines will give the proportional mortality corresponding to equal times of life. The half-lives are found from the intersections of the curves with the value 0·693, and in general, the time corresponding to a fraction $1/F$ from the intersections with the value $\log_e F$.

TABLE I.

| Strain. | Half-life. | Expectation of life. |
|---|---|---|
| Bittner-A | 12·40 ± 0·16 | 12·57 |
| Marsh-A | 14·49 ± 0·17 | 14·70 |
| dba | 10·83 ± 0·08 | 11·04 |
| A-FC | 12·08 ± 0·31 | 12·22 |
| A-RO | 12·90 ± 0·32 | 12·79 |
| C3H | 11·40 ± 0·36 | 11·78 |
| D-stock | 11·20 ± 0·07 | 11·37 |

The standard half life with its sampling error and the expectation of life of each of the seven strains which have been dealt with in this paper are given in Table I. It is interesting to compare these with the half-lives for the offspring of the two reciprocal crosses between C57 Black and dba. They are : 25·6 for the cross with a dba mother, and 37·8 for the reciprocal.

From a study of the $\mu(x)$ graphs it is obvious that the differences in the form of the curves from strain to strain make strict comparisons of tumour susceptibility, of the same kind as are made in biological assay, impossible. Future work will probably reveal some of the factors which determine the shape of the curves, but in the meantime it should be emphasized that susceptibility to mammary tumour is determined in a different way in each strain, though the similarity of the $\mu(x)$ curves of different groups of mice of the same pure line does enable more exact comparisons to be made in this case. The main use of the $\mu(x)$ curves here has been to remove an outstanding source of variation between strains, namely, the non-tumour mortality. The results suggest that it is worth while to make a similar investigation using the function $\mu(x)$ whenever such life tables are available.

### SUMMARY.

The use of an actuarial function, descriptive of tumour susceptibility, is discussed in relation to the published statistics of some cancer-prone pure lines of mice.

The general problem of assessing tumour susceptibility and some measures of it are discussed.

I must record my thanks to Professor J. B. S. Haldane, who first suggested that the $\mu(x)$ curves could be used for the investigation of tumour susceptibility, and who provided me with the method of calculation given in Appendix I. To Dr. C. A. B. Smith I owe the maximum likelihood solution of Appendix II, and both to him and to Professor Haldane I am grateful for much good advice and helpful discussion.

### REFERENCES.

BRYAN, W. R., AND SHIMKIN, M. B.—(1941) *J. Nat. Cancer Inst.*, **1**, 807.
DUBLIN, L. I., AND LOTKA, A. J.—(1920, 1921) 'Stat. Bull. Metropolitan Life Insurance Co.,' Vols. 1 and 2.
IRWIN, J. O.—(1935) *J.R. Stat. Soc.*, **98**, 83.
*Idem* AND GOODMAN, N.—(1946) *J. Hyg., Camb.*, **44**, 362.
KARN, M. N.—(1933) *Biometrika*, **25**, 91.
LEA, D. E.—(1945) *Cancer Res.*, **5**, 633.
MURRAY, W. S., AND BITTNER, J. J.—(1936) *Amer. Nat.*, **70**, 443.
*Idem* AND HOFFMAN, J. G.—(1941) *Cancer Res.*, **1**, 298.
TWORT, C. C., AND TWORT, J. M.—(1930) *J. Hyg., Camb.*, **29**, 373.—(1933) *Amer. J. Cancer*, **17**, 293.

### APPENDIX I.

### METHODS OF ESTIMATING $\mu(x)$.

The formula used for calculating the values of $\mu(x)$ was

$$\mu(x) = \frac{\frac{1}{2}(M(x-1) + M(x))}{l(x) + \frac{1}{6}\Delta^2 l(x-1)}. \quad . \quad . \quad . \quad . \quad (1)$$

Where $M_x$ = cancer deaths between ages $x$ and $x + 1$.
$l(x)$ = number of animals alive at age $x$.

There is a slight bias in the estimate of $\mu(x)$ given by this formula which tends to be too low. As originally derived the value given by expression (1) could be improved by successive approximations. This process is too laborious to be practical, and if the values of $\mu(x)$ were required with more accuracy it would be preferable to calculate

$$l(x + \tfrac{1}{2})\, \mu(x + \tfrac{1}{2}) = (M(x) - \tfrac{1}{24}\Delta^2 M(x-1)). \quad . \quad . \quad (2)$$

Then $(x + \frac{1}{2})$ can be found from the $l(x)$ values of the life table by interpolation and thence $\mu(x + \frac{1}{2})$ by division; the rough formula to which this process leads is

$$\mu(x + \tfrac{1}{2}) = \frac{M(x)}{\frac{1}{2}(l(x) + l(x-1))}. \quad . \quad . \quad . \quad . \quad (3)$$

This formula or

$$\mu(x) = \frac{\frac{1}{2}(M(x-1) + M(x))}{l(x)}$$

are perfectly adequate approximations for all ordinary purposes.

The relationships between the indices discussed in the paper and the $\mu(x)$ curves are worth noting.

(1) *Percentage of tumour deaths.*

Let $\mu(x)$ = total force of mortality.
$\mu'(x)$ = cancer force of mortality.
$\mu''(x)$ = non-cancer force of mortality.

Then proportion of tumour deaths is

$$P = \int_0^\infty \mu'(x)\, l(x)\, dx \Big/ \int_0^\infty \mu(x)\, l(x)\, dx$$

$$= \int_0^\infty \frac{\mu'(x)}{\mu(x)}\, \mu(x)\, l(x)\, dx \Big/ \int_0^\infty \mu(x)\, l(x)\, dx.$$

and is therefore an average of the ratio of cancer $\mu(x)$ to the total $\mu(x)$ weighted by the deaths at age $x$.

(2) *Expectation of life.*

This is given by

$$e = \int_0^\infty l(x)\, dx.$$

Now

$$\int_0^\infty l(x)\, \mu'(x)\, dx = 1$$

in a table of mortality subject to $\mu'(x)$ alone.*

So the reciprocal of the expectation of life is equivalent to the average value of $\mu(x)$ weighted by the probability of living to age $x$. It is in fact the death rate that would be observed in a stationary population exposed to the table of mortality under consideration.

(3) *Monthly death rates and half life.*

Let $P_x$ = probability of surviving cancer death within one month at age $x$.

Then $P_x = \exp. - \left[\int_x^{x+1} \mu'(x)\, dx\right]$ in a life table subject to cancer mortality alone.

$$\therefore \log. P_x = -\int_x^{x+1} \mu'(x)\, dx.$$

$$\therefore -\sum_{x=0}^{t} \log. p_x = \Sigma \log. \left(\frac{1}{p_x}\right) = \int_0^{t+1} \mu(x)\, dx.$$

* When no other sources of mortality exist then Iball's index of carcinogenicity is equivalent to the number obtained from : $\int_0^\infty l(x)\, \mu'(x)\, dx \Big/ \int_0^\infty l(x)\, dx.$

Now probability of reaching age $x$ is exp. — $[\int_o^x \mu'(x)\, dx]$, and since exp. — $[\log._e 2] = \frac{1}{2}$, the value of $t$ at which $\sum_o^t \log. \left(\frac{1}{p_x}\right) = \log._e 2 = 0\cdot693$ gives the half life.

(4) *Modified expectation of life.*

The number obtained by taking the reciprocal of the sum $\Sigma$ log. $(1/p_x)$ for the whole table is of interest since it gives an estimate of the expectation of life.

Suppose the table extends over ages from $o - t$ so that the sum of probabilities is

$$\sum_o^{t-1} \log. (1/p_x) = \Sigma_t,$$

then we can write the reciprocal of this sum

$$\frac{1}{\Sigma_t} = \frac{1}{\int_o^t \mu(x)\, dx}.$$

Now

$$\int_o^t \mu'(x)\, l(x)\, dx = 1$$

if there is no cause of death but cancer.

So that the reciprocal is a weighted mean of the $\mu(x)$'s. Multiplying this by $t$, the interval over which the table extends, gives an estimate of the expectation of life. This estimate is rather lower than that given by the correct method of calculation since the values of $l(x)$ are weighted in favour of the low values at the higher ages where the $\mu(x)$'s are large. It might be preferable to use this modified life expectation instead of the half-life as it takes into account all the data and can be given a standard error. The standard error of $\Sigma_t$ is calculated as for the 50 per cent value (Appendix II), the sum being taken over the whole table ; then if the modified expectation of life is given by

$$E' = t/\Sigma_t.$$

Then the variance of $E'$ is approximately

$$\text{Var. } (E') = \frac{t^2}{\Sigma^4_t} \text{ Var. } (\Sigma_t)$$

and its standard error is

$$\text{S.E. } (E') = \frac{t}{\Sigma^2} \text{ S.E. } (\Sigma_t).$$

### APPENDIX II.

### THE MAXIMUM LIKELIHOOD METHOD OF ESTIMATING SPECIFIC MORTALITIES.

Let $P_x$ = probability of surviving death from cancer for one month at age $x$, if cancer alone were effective.

$P'_x$ = similar probability for causes other than cancer.
$d_x$ = observed cancer deaths between ages $x$ and $x + 1$.
$d'_x$ = observed non-cancer deaths between ages $x$ and $x + 1$.
$1_x$ = number alive at age $x$.

Then probability of survival = $P_x\, P'_x$ and probability of death from cancer = $(1 - p_x)\, p'_x$, probability of death from other causes = $(1 - p'_x)\, p_x$.

There is another term $(1 - p_x)(1 - p'_x)$ which it is logical to divide equally between the two probabilities of death since it measures the probability of "death from both causes." So we have

$$\text{Probability of death from cancer} = (1 - p_x)p'_x + \frac{(1 - p_x)(1 - p'_x)}{2}$$

$$= \tfrac{1}{2}(1 - p_x)(1 + p'_x).$$

Probability of death from other causes = $\tfrac{1}{2}(1 - p'_x)(1 + p_x)$.

The likelihood of the observed results at age $x$ is proportional to

$$[(1 - p_x)(1 + p'_x)]^{dx}\, [1( - p'_x)(1 + p_x)]^{d'x}\, [p_x p'_x]^{lx+1}$$

and the logarithm of the likelihood is, apart from a constant,

$$L = d_x \log.\,(1 - p_x) + d_x \log.\,(1 + p_x) + d'_x \log.\,(1 - p'_x) + d'_x \log.\,(1 + p_x) + l_{x+1} \log.\,p_x + l_{x+1} \log.\,p'_x.$$

Differentiating, we have

$$-\frac{d_x}{1 - p_x} + \frac{d'_x}{1 + p_x} + \frac{l_{x+1}}{p_x} = \frac{\partial L + 1}{\partial p_x} \quad . \quad . \quad . \quad (1)$$

and

$$\left(\frac{d_x}{(1 - p_x)^2} + \frac{d_x}{(1 + p_x)^2} + \frac{l_{x+1}}{p_x^2} = \frac{\partial^2 L}{\partial p_x^2}\right) = -\frac{1}{\text{Var.}\,(p_x)} \quad . \quad . \quad (2)$$

Equating the right-hand side of equation (1) to zero we obtain the equation of estimation of $p_x$. This may be solved as a quadratic or by successive approximations.

The method of obtaining the half life is to find a value of $x$ such that

$$P_0 P_1 \ldots p_x = \tfrac{1}{2} \text{ or } \log.\left(\frac{1}{p_1}\right) + \log.\left(\frac{1}{p_2}\right) + \ldots \log.\left(\frac{1}{p_x}\right) = \log.\,2.$$

In practice as the values of $P_x$ are given at monthly intervals the equation will not be satisfied for any integral value of x and the half life time must be found by interpolation.

If the half life is determined from the sum of the log. $P_x$'s, then it is possible to assign a standard error to the sum, for

$$\text{Var. (log. } p_x) = \frac{1}{p_x^{\ 2}} \text{ Var. } (p_x)$$

and the variance of the sum is equal to the sum of the variances since the $p'_x$'s are independent.

If we call this sum $\Sigma$ then the variance of the half life will be given by

$$\text{Var. } (\Sigma) = \left(\frac{\partial \Sigma}{\partial x}\right) \text{Var. } (x),$$

and since

$$\frac{\partial \Sigma}{\partial x} = \text{log. } p_x \text{ (approximately)},$$

$$\text{Var. } (x) = \frac{\text{Var. } (\Sigma)}{(\text{log. } p_x)^2}$$

and the standard error of $x$ is

$$\frac{\text{S.E. } (\Sigma)}{\text{log. } P_x}.$$

The method of Dublin and Lotka assumes, as before, that the probability of living one month at age $x$ is given by $P_x = P_x\ P'_x$. If $Q_x$ is the probability of dying within one month at age $x$ in an ordinary life table, with similar meanings for $q_x$ and $q'_x$, then

$$(1 - Q_x) = (1 - q_x)\,(1 - q'_x).$$

Therefore

$$q_x = \frac{(Q_x - q'_x)}{(1 - q'_x)}.$$

These values of $q_x$ may be used to build up a new life table from which non-cancer mortality has been eliminated.

The values obtained by this method were used as first approximations to the maximum likelihood solution and a better approximation found by iterating. This was convenient as they are close to the likelihood values and had been calculated previously. As a general practice it would be better to solve the quadratic, which can be done quite rapidly and systematically using Barlow's Tables. Dublin's and Lotka's formula was put forward independently in toxicological work by Abbot and used by him and Tattersfield and Morris. An account of its use is given by Finney (' Probit Analysis,' Cambridge, 1947).